# Acoustic analysis of vowel production in adults with autism spectrum disorder: beyond the notion of a unitary autistic voice

**DOI:** 10.3389/fpsyg.2026.1854297

**Published:** 2026-07-13

**Authors:** Georgios P. Georgiou, Maria Paphiti

**Affiliations:** 1 Department of Languages and Literature, University of Nicosia, Nicosia, Cyprus; 2 Phonetic Lab, University of Nicosia, Nicosia, Cyprus; 3 Department of Health Sciences, European University Cyprus, Nicosia, Cyprus

**Keywords:** acoustic analysis, adults, autism spectrum disorder, Cypriot Greek, vowel

## Abstract

**Introduction:**

Speech in autism spectrum disorder (ASD) carries distinctive acoustic signatures that can offer valuable insight into the nature of autistic communication and its identification. Among these, vowel production remains insufficiently understood, despite its central role in speech intelligibility. This study investigates whether ASD is associated with systematic differences in vowel production.

**Methods:**

Eighteen Cypriot Greek adults with ASD and 18 peers with neurotypical development (ND), comparable in age, gender, education, nonverbal IQ, and verbal fluency, completed a controlled reading task. Participants produced disyllabic pseudowords embedding the five Greek vowels across four stress-syllable contexts. Acoustic analyses measured vowel-space organization, static spectral properties (F0, F1, F2, F3), dynamic trajectories (ΔF0, ΔF1, ΔF2, ΔF3), duration, and voice-quality indices (jitter, shimmer, harmonic-to-noise ratio [HNR], intensity). Bayesian models were used to evaluate group and vowel-specific differences.

**Results:**

The results revealed a larger vowel-space area and greater vowel-space dispersion in the ASD group relative to the ND group, indicating a more expanded and dispersed acoustic vowel system. Group differences in individual acoustic measures were mostly selective rather than global: the clearest effects emerged in vowel-specific patterns of pitch, formants, and some dynamic formant measures. By contrast, duration, jitter, shimmer, and intensity did not show robust vowel-specific group differences. Among voice-quality measures, HNR showed the most consistent group difference across individual vowels, with ASD speakers showing higher HNR across all vowels.

**Discussion:**

These findings challenge the notion of a single, uniform autistic voice, instead demonstrating that autism-related speech differences are multidimensional, vowel-specific, and language-sensitive. They therefore underscore the critical importance of segment-focused, cross-linguistically grounded approaches for advancing theory, assessment, and future speech-based identification in autism research.

## 1 Introduction

Autism spectrum disorder (ASD) is a neurodevelopmental condition characterized by persistent differences in social communication and social interaction, together with restricted and repetitive patterns of behavior, interests, or activities (Centers for Disease Control and Prevention, 2025; Kamp-Becker, 2024). Current diagnostic frameworks further emphasize that ASD is highly heterogeneous in its clinical presentation, developmental trajectory, and functional impact across individuals (Lord et al., 2018; Masi et al., 2017). Because no single biological marker is sufficient for diagnosis, behavioral manifestations remain central to clinical identification and characterization (Cortese et al., 2025). Among these, speech is particularly important, as it is one of the most immediate and socially consequential channels through which communicative differences are perceived in both clinical and everyday settings (Diehl et al., 2008; Trayvick et al., 2024). Accordingly, vocal production has become a key domain in research on autism, especially in efforts to understand how autism-related differences are expressed in observable communicative behavior.

Clinical descriptions have long suggested that some autistic individuals produce speech that is perceived as acoustically distinctive, often in terms of atypical prosody, unusual intonation, altered rhythm, or differences in voice quality (Godel et al., 2023; McCann and Peppé, 2003). Acoustic phonetics offers a useful framework for investigating such impressions because it allows speech to be analyzed through measurable properties of the acoustic signal. In autism research, this has motivated growing interest in whether ASD is associated with reliable differences in core dimensions of speech production, including prosodic control, phonation, articulation, and temporal organization (Briend et al., 2023; Lau et al., 2022). These dimensions can be examined through measures such as fundamental frequency (F0), vowel formants, within-vowel spectral change, duration, and voice-quality indices, including jitter, shimmer, harmonic-to-noise ratio (HNR), and intensity (Kent and Vorperian, 2018; Lee et al., 2023; Van Santen et al., 2009). Because these measures can be extracted from short and controlled speech samples, they provide a means of studying speech production while minimizing the lexical, pragmatic, and interactional demands that characterize spontaneous conversation (Georgiou and Kaskampa, 2024). In this way, acoustic analysis may contribute to the more precise characterization of autism-related speech patterns and, potentially, to future research examining whether some acoustic patterns may prove useful for screening-oriented applications.

### 1.1 Differences between individuals with ASD and ND in various acoustic measures

Vowel production in ASD has frequently been characterized as atypical on perceptual grounds, but the acoustic-phonetic literature has increasingly specified the signal dimensions on which ASD and ND speakers converge or diverge.

F0-related differences have been examined less often at the vowel level than at the broader prosodic level, in which ASD individuals appear to have higher mean F0, wider F0 range, and greater F0 variability on average than ND controls (Asghari et al., 2021). At the level of vowel F0, the evidence does not currently support a robust, consistent ASD–ND difference. The clearest direct comparison comes from Arciuli and Bailey (2019), who examined lexical-stress production in autistic and neurotypical Italian-speaking children and measured F0 on the first two vowels of target trisyllabic words produced in a picture-naming task. They found that the two groups showed a similar magnitude of stress contrastivity for vowel F0, indicating a null group difference in this vowel-level pitch measure. In contrast, Mohanta and Mittal (2022), using a controlled word production task, found that children with ASD showed higher mean F0 and greater F0 variability than neurotypical children across the five English vowels. There is also related evidence from tone-language research. Xu et al. (2022) compared Mandarin-speaking autistic and neurotypical children and found atypical patterns in the height and shape of lexical tones. Similarly, Guo et al. (2022) reported that F0 in Mandarin tone production was higher in children with ASD than in ND peers across prosodic positions and lexical tones.

With respect to formants and vowel-space measures, the literature suggests that ASD–ND differences can emerge, although the direction of effects is not consistent across studies. Lyakso et al. (2016) reported larger vowel formant triangles in Russian-speaking autistic children than in neurotypical peers, consistent with a more expanded vowel-space pattern. Mohanta and Mittal (2022) reported higher F1, F2, F3, and F5 values across all five target English vowels, as well as higher F4 values for three vowels, in autistic children than in neurotypical children, interpreting these differences as evidence of atypical vowel production. In contrast, Sadiq et al. (2025) reported significantly lower F1 and F2 in the ASD group consisting of Urdu-speaking Pakistani children; however, they used a sustained vowel elicitation method. In another line of evidence, Kissine et al. (2021) found that French-speaking autistic adults showed reduced dispersion in F1–F3 space during native vowel production and less variable imitation of unfamiliar vowel-like targets, a pattern interpreted as greater articulatory stability but reduced phonetic flexibility. Finally, Hong et al. (2023) found no clear ASD–ND difference in vowel-formant entrainment among second language English-speaking children, suggesting that some aspects of vowel adaptation may remain intact under controlled interactional conditions. Overall, these findings suggest that ASD–ND differences in vowel production can be reflected in formant values and vowel-space organization, but that the sign of the effect varies across studies, ranging from higher/lower formants or expanded vowel space to reduced dispersion, with some studies also reporting no reliable group difference in interactive adaptation.

Evidence on duration at the vowel level is limited and tends toward null findings rather than a clearly consistent atypical pattern. In the lexical-stress study by Arciuli and Bailey (2019), vowel duration was measured directly, and the ASD and neurotypical groups showed a similar magnitude of stress contrastivity. In addition, Sadiq et al. (2025) found no significant group difference in duration. Together, these findings suggest that vowel timing may often be comparable to neurotypical production under structured elicitation conditions, although broader speech-timing studies have reported occasional duration differences in other task contexts (Maffei et al., 2023). For jitter, shimmer, HNR, and intensity, the vowel-focused literature is smaller. For example, Kissine et al. (2021) reported lower jitter and lower shimmer in autistic adults, consistent with greater stability of voicing during vowel production. However, this pattern is not universal, and broader reviews note that shimmer differences are often absent or inconsistent (Bone et al., 2014) and that perturbation-related measures are especially sensitive to vowel type, elicitation conditions, and recording quality (MacCallum et al., 2011; Yousef and Hunter, 2024). Evidence for HNR and intensity at the vowel level is more limited and less consistent. Some studies (e.g., Guo et al., 2022) report that HNR does not appear as a vowel-level marker of ASD, although Briend et al. (2023) found higher HNR in children with ASD during a repetition task using nonwords composed of the vowels [i a u]. Intensity findings are likewise limited: Arciuli and Bailey (2019) found largely similar group performance between ASD and ND children overall, and, similarly, Sadiq et al. (2025) found no significant global group intensity difference between ASD and ND individuals in this measure.

Table 1 shows that previous ASD acoustic studies vary substantially in developmental stage, language, elicitation method, and acoustic parameters examined. These differences may partly explain why findings are inconsistent across studies. Sustained-vowel tasks may emphasize phonatory stability and voice-quality measures, lexical-tone tasks emphasize F0 control, nonword repetition engages auditory–motor sequencing, entrainment paradigms assess adaptive phonetic alignment, and picture-naming or word-production tasks may involve lexical retrieval and stress-related demands. Therefore, differences across studies may reflect not only ASD–ND differences in speech production, but also task demands, language-specific phonetic structure, developmental stage, and the specific acoustic variables selected for analysis.

**Table 1:** Summary of studies examining acoustic measures in ASD across languages, populations, and methodologies.

Authors	Population	Language	Methodology	Acoustic parameters
Arciuli and Bailey (2019)	Children with ASD and TD children	Italian	Picture-naming lexical-stress task	Vowel F0, vowel duration, lexical-stress contrastivity
Asghari et al. (2021)	Review/meta-analysis; mixed ASD populations	Mixed	Systematic review/meta-analysis	Mean F0, F0 range, F0 variability, duration/prosodic measures
Bone et al. (2014)	Children with ASD	English	Spontaneous speech during ASD assessment/interlocutor interaction	Prosodic and voice-quality measures, including pitch, timing, intensity, HNR
Briend et al. (2023)	Children with ASD, TD children, and clinical controls	French	Nonword repetition task; machine-learning classification	HNR, intensity, F0, formant- and perturbation-related measures
Guo et al. (2022)	Children with ASD and TD children	Mandarin	Lexical-tone production; random forest classification	Voice-quality/acoustic parameters during tone production
Hong et al. (2023)	Children with ASD and TD children	English as L2	L2 human–robot interaction; phonetic entrainment task	Vowel-formant entrainment, mean F0 entrainment, F0-range entrainment
Kissine et al. (2021)	Adults with ASD and NT adults	French	Native vowel production and imitation of unfamiliar vowel-like targets	F1–F3 dispersion/variability, F0, jitter, shimmer
Lyakso et al. (2016)	Children with ASD and TD children	Russian	Acoustic analysis of child speech	Vowel formants, vowel formant triangles, pitch/formant-related features
Mohanta and Mittal (2022)	Children with ASD and non-ASD children	English	Controlled speech-sound production and classification	F0, F0 variability, vowel formants, acoustic classification features
Sadiq et al. (2025)	Children with ASD and ND children	Mainly Urdu	Sustained-vowel elicitation; acoustic/perceptual voice analysis	F1, F2, F3, duration, intensity, F0/global acoustic measures
Xu et al. (2022)	Children with ASD and TD children	Mandarin	Lexical-tone production task	Tone height, tone shape, F0 contours

### 1.2 Challenges and gaps in the literature

Although many studies report acoustic differences between ASD and ND speakers, the evidence base remains difficult to consolidate into a stable profile. Reviews and meta-analyses suggest that ASD-related differences can be detected in vocal production, especially in prosodic dimensions, but they also show substantial variation across studies in both effect size and direction (Asghari et al., 2021; Fusaroli et al., 2017; Ma et al., 2024). As a result, the field has not yet established which acoustic measures can be treated as robust, generalizable markers of ASD rather than task- or sample-specific correlates. A major reason for this uncertainty is methodological variability. Measures often interpreted as indices of phonatory stability or voice quality are highly sensitive to recording conditions, segmentation practices, analysis settings, and signal quality (Carson et al., 2003; Dejonckere et al., 2012; Manfredi et al., 2011; Titze, 1995; Vogel et al., 2009). Even under typical clinical conditions, these measures can vary systematically with vowel identity, fundamental frequency, and vocal intensity (Brockmann et al., 2011). In addition, acoustic differences observed in conversational or narrative speech are often difficult to interpret because such tasks engage not only speech production but also lexical retrieval, discourse organization, pragmatic adaptation, affective stance, and interactional coordination (Ma et al., 2024; Patel et al., 2020). Consequently, apparent group differences may sometimes reflect elicitation, task demands, or processing decisions rather than stable differences in speech production itself. This has motivated the use of more structured elicitation paradigms, which can reduce some sources of task-related variability and allow more controlled assessment of speech output (Diehl and Paul, 2009, 2013; McCann and Peppé, 2003). Such designs are especially useful for isolating segmental and phonatory properties more precisely.

Furthermore, the existing literature is heavily weighted toward child samples, with comparatively less evidence from adolescents and adults. This makes it difficult to determine whether reported acoustic characteristics reflect stable ASD-related patterns or developmental differences that may change with age. This developmental imbalance is important because vowel-space measures are not developmentally static. In typical development, children’s vowels generally show higher formant frequencies and larger or less consistent vowel spaces than adults’ vowels, reflecting vocal-tract growth, anatomical maturation, and increasing speech-motor stability; formant frequencies and vowel-space area tend to decrease and stabilize across childhood and adolescence (Meister and Meister, 2021; Pettinato et al., 2016; Vorperian and Kent, 2007). Comparable developmental evidence in ASD is much more limited, especially for longitudinal change from childhood to adulthood. Existing studies suggest that autistic children may show differences in speech-motor control, phonetic stability, or vowel-space organization, but it remains unclear whether these patterns persist, attenuate, or reorganize in adulthood. Therefore, findings from child ASD cohorts cannot be directly extrapolated to autistic adults. In the present study, child findings are used only as developmental background and hypothesis-generating evidence.

Another limitation concerns language coverage. Much of the available evidence comes from English-speaking samples, even though the acoustic realization of prosody and segmental structure is shaped by language-specific phonological systems and culturally patterned speech norms (Fusaroli et al., 2022; Gussenhoven, 2002). Cross-linguistic work has already shown that not all potentially relevant acoustic dimensions behave similarly across languages. For example, Lau et al. (2022) found that some rhythm-related features generalized more readily across English and Cantonese, whereas intonation-related features were more language-dependent. This suggests that acoustic correlates of ASD cannot simply be assumed to transfer unchanged from one language to another. These concerns are especially relevant for Greek, and particularly Cypriot Greek, which remains largely absent from the autism acoustics literature despite having a well-described vowel system (Georgiou, 2023a; Georgiou and Savva, 2026). Existing Greek and Cypriot Greek ASD research (e.g., Charalambous Darden et al., 2016; Christopoulou et al., 2021; Zarokanellou et al., 2025) has focused more on broader language abilities than on acoustic-phonetic analyses of speech production. To date, the only phonetics-oriented study in this area appears to be Georgiou (2023b), which examined vowel identification in normal versus whispered speech and therefore addressed speech perception rather than production. As a result, there is still very limited evidence on how ASD and ND speakers of Cypriot Greek, or Greek in general, differ in vowel production, voice quality, or vowel-space organization.

The present study responds to these issues by focusing on controlled vowel production in Cypriot Greek using phonotactically legal pseudoword frames. This design minimizes lexical and pragmatic confounds, allows consistent segmentation of vowel intervals, and enables ASD–ND comparisons across multiple acoustic dimensions within a single adult sample and task context. Rather than providing a definitive resolution of the mixed literature, the study contributes targeted language-specific evidence from an underrepresented variety and helps clarify how vowel-level acoustic patterns appear under controlled adult production conditions.

### 1.3 This study

The present study examines whether speakers with ASD and ND peers differ in vowel production across a broad set of acoustic dimensions, including static spectral measures (F0, F1, F2, F3), dynamic spectral measures (ΔF0, ΔF1, ΔF2, ΔF3), temporal structure (duration), and voice-quality measures (jitter, shimmer, HNR, and intensity). The study is original in providing a controlled, segment-focused acoustic analysis of Cypriot Greek, a linguistic variety that remains largely absent from the autism acoustics literature despite the field’s increasing recognition that acoustic markers may not generalize straightforwardly across languages. By focusing on a well-defined five-vowel system in a tightly controlled elicitation paradigm, the study addresses an important empirical gap while extending autism speech research beyond the predominantly English-based evidence base.

The contribution of the study is therefore twofold. First, it provides language-specific baseline evidence for Cypriot Greek, where production-based acoustic work on ASD is currently very limited. Second, it offers a more fine-grained account of ASD–ND differences by examining not only overall group effects, but also vowel-specific contrasts within a Bayesian modelling framework. In this way, the study moves beyond broad claims of “atypical prosody” or “atypical voice” and instead asks which specific acoustic dimensions and which specific vowels differentiate the two groups when communicative demands are minimized and measurement conditions are tightly controlled. This is an important step toward clarifying the mixed findings in the literature and toward evaluating which acoustic features may prove most robust for future descriptive and stratificational work in ASD, and for possible future screening-oriented research pending replication and validation.

Based on the previous literature, several hypotheses were formulated. First, because prior studies have reported ASD–ND differences in vowel-space organization and formant structure (e.g., Kissine et al., 2021; Lyakso et al., 2016; Mohanta and Mittal, 2022), we expected group differences to emerge in vowel-space measures and in at least some formant-related acoustic dimensions, although the direction of these effects was treated cautiously given the inconsistency of previous findings. Second, based on previous reports of limited or inconsistent ASD–ND differences in duration, jitter, shimmer, and intensity, we expected these measures to show weaker or less consistent group effects. Third, because previous findings suggest that ASD-related acoustic differences may be vowel-specific rather than global, we expected any observed group differences to be distributed unevenly across vowels and acoustic dimensions rather than appearing uniformly across the vowel system.

## 2 Methodology

### 2.1 Participants

Thirty-six individuals with Cypriot Greek as their native language participated in the study. The experimental group included 18 participants (15 males/3 females) with a diagnosis of high functioning ASD documented in official clinical records (*M*_diagnosis age_ = 9.88; SD = 10.59). Diagnoses had been made by experienced clinicians, neurologists, and psychiatrists, in accordance with the Diagnostic and Statistical Manual of Mental Disorders, Fifth Edition (DSM-5) criteria. Participants in the ASD group were aged 18–40 years and were recruited from specialized autism centers in Cyprus and, where eligible, from adult students in upper secondary schools. Exclusion criteria included a history of intellectual disability and co-occurring language disorder. Available clinical records indicated that some participants in the ASD group reported co-occurring diagnoses, but none had a documented language disorder. Some participants also reported current or previous medication use, while all reported current or previous therapeutic support. Because co-occurring conditions are common in autistic adults, these participants were retained to preserve ecological validity. However, detailed information regarding ASD support level, specific psychiatric comorbidities, and speech-language history was not consistently available for all participants. The control group comprised 18 participants (15 males/3 females) with ND, aged 18–42 years, recruited across Cyprus. These participants had no reported history of neurodevelopmental, language, cognitive, or behavioral disorders.

To characterize the sample, participants completed brief background measures of empathy, autistic traits, verbal fluency, and nonverbal reasoning. Empathy was assessed with the Greek adaptation of the Empathy Quotient (EQ; Baron-Cohen and Wheelwright, 2004; Pehlivanidis et al., 2021), a self-report measure in which higher scores indicate greater empathy. Autistic traits were assessed with the Greek version of the Autism-Spectrum Quotient (AQ; Baron-Cohen et al., 2001), with higher scores indicating greater autistic trait expression. Verbal fluency was measured using the Greek adaptation of the fluency test (Kosmidis et al., 2004), including both semantic and phonemic tasks, from which total word production, mean cluster size, and number of switches were calculated. Nonverbal reasoning was assessed with Raven’s Progressive Matrices, Second Edition (Raven et al., 2018), using Sets B–E, with total correct responses used as the score.

Groups were comparable for linguistic, cognitive, social, and other variables. More specifically, the ASD and ND groups did not differ significantly in terms of chronological age, nonverbal IQ, education, and gender distribution. They also did not differ in any measures of the fluency test; semantics test: total word production (WP), (b) mean cluster size (SC), reflecting semantic or phonemic grouping of responses, and (c) number of switches (SW); phonemic test: WP, SC, SW. Furthermore, as expected, the two groups differed significantly in Empathy Quotient (EQ) scores and Autism–Spectrum Quotient (AQ) scores, with the ASD group showing lower EQ scores and higher AQ scores, confirming autistic traits. All participants reported normal or corrected–to–normal vision and hearing. Participant demographics and test scores are summarized in Table 2.

**Table 2:** Participants’ demographics and scores in the tests.

Variable	ASD (*n* = 18)	ND (*n* = 18)	Statistical test results
Sex, male/female, *n*	15/3	15/3	*χ*^2^ = 0, *df* = 1, *p* = 1
Age, years (SD)	24.1 (6.43)	24.5 (7.11)	*t* = −0.17, *df* = 34, *p* = 0.86
EQ score (SD)	29.7 (12.4)	43.1 (10.2)	*t*(34) = −3.55, *p* = 0.001
AQ score (SD)	29.6 (6.49)	14.8 (5.96)	*t*(34) = 7.11, *p* < 0.001
Nonverbal IQ raw score (SD)	26.6 (3.91)	28.9 (3.35)	*t* = −1.97, *df* = 34, *p* = 0.06
Education, median IQR^*^	3	3	*W* = 149, *p* = 0.68
Semantics test—WP score (SD)	49.56 (7.79)	53.33 (5.84)	*t* = −1.65, *df* = 34, *p* = 0.11
Semantics test—SC score (SD)	7.44 (3.57)	6.94 (2.90)	*t* = 0.46, *df* = 34, *p* = 0.65
Semantics test—SW score (SD)	34.61 (7.70)	40.44 (12.18)	*t* = −1.72, *df* = 34, *p* = 0.09
Phonemic test—WP score (SD)	30.67 (6.24)	36.11 (9.92)	*t* = −1.97, *df* = 34, *p* = 0.06
Phonemic test—SC score (SD)	1.89 (1.37)	2.44 (2.48)	*t* = −0.83, *df* = 34, *p* = 0.41
Phonemic test—SW score (SD)	26.44 (3.75)	29.56 (6.17)	*t* = −0.83, *df* = 34, *p* = 0.08

* Highest educational attainment; UNESCO Institute for Statistics (2012).

Before participation, all individuals (and guardians where applicable) received written information about the study procedures and their rights, and provided informed consent in accordance with the Declaration of Helsinki.

### 2.2 Materials and tools

The materials consisted of disyllabic pseudowords presented in four contexts: /ˈsVsa/, /sVˈsa/, /ˈVsa/, and /Vˈsa/ (V = vowel). Each context targeted the five Greek vowels /i e a o u/. The target vowels were always those found in the first syllable. All stimuli were constructed to conform to Greek phonotactic constraints and to be word-like while remaining semantically meaningless. The four contexts were selected to allow the target vowels to be elicited in a range of controlled phonetic environments (the presence vs. absence of an onset consonant and stressed vs. unstressed vowel). The fricative /s/ was chosen as a stable consonantal frame with relatively minimal coarticulatory influence on adjacent vowels, enabling clearer assessment of vowel realization. Comparing /sVsa/ with /Vsa/ contexts makes it possible to control for the effect of onset consonants and stress on vowel production. All contexts are phonotactically well-formed in Greek and word-like without carrying lexical meaning, ensuring naturalistic articulation while avoiding lexical or semantic confounds.

### 2.3 Procedure

#### 2.3.1 Production test

Each participant completed the production task individually in a quiet room. Participants were seated at a desk facing a PC monitor at an approximate distance of 50–70 cm. Stimuli were presented in Standard Modern Greek orthography, and participants were instructed to read each word aloud at a comfortable speaking rate and volume. Recordings were made using a Zoom H5 digital recorder (44.1 kHz, 16-bit; WAV format). The recorder placement and mouth-to-recorder distance were kept constant across participants, and input levels were adjusted before testing to avoid clipping. Each participant produced 80 phrases (5 vowels × 4 contexts × 4 repetitions), yielding 2,880 phrases overall. Items were randomized within each repetition block. A brief familiarization phase preceded the experiment to ensure accurate reading; practice items were not included in the analysis. Productions containing misreadings, disfluencies, or excessive noise were re-recorded when possible or excluded. Participants were allowed short breaks of up to 5 min between repetition blocks.

#### 2.3.2 Feature extraction

The target words produced by the speakers were extracted and processed in Praat (Boersma and Weenink, 2026). Tokens were inspected auditorily and visually using waveforms and spectrograms. When a word was mispronounced or produced with hesitation, participants were asked to repeat it. Vowel boundaries were manually segmented in Praat on the basis of waveform and spectrographic information. Segmentation was performed with knowledge of the participant group. However, to assess the reliability of vowel boundary marking, both inter-rater and intra-rater reliability checks were conducted. Vowel onset was identified as the beginning of vocalic periodicity following the offset of frication noise in /ˈsVsa/ and /sVˈsa/ contexts, and as the onset of vocalic periodicity in /ˈVsa/ and /Vˈsa/ contexts. Vowel offset was identified as the end of vocalic periodicity before the following consonant.

Formant and pitch measurements were extracted using Praat scripts. Formant extraction was conducted using the Burg method with a time step of 0.01 s, a maximum of five formants, a maximum formant ceiling of 5,500 Hz, a window length of 0.025 s, and pre-emphasis from 50 Hz. F0 was extracted with a pitch time step of 0.01 s and a pitch range of 75–300 Hz. F0, F1–F3, and intensity were measured at the midpoint of each vowel segment to minimize coarticulatory effects, whereas vowel duration corresponded to the full duration of the segmented vowel interval. Formant tracks and extracted values were inspected for quality. For voice-quality measures, Praat’s jitter (local), shimmer (local), and harmonicity (cc) functions were used. Jitter and shimmer were computed from PointProcess objects generated with a pitch range of 75–600 Hz. Intensity was extracted using a minimum pitch setting of 100 Hz, and HNR was calculated using the cross-correlation harmonicity method with a 0.01 s time step, 75 Hz minimum pitch, 0.1 silence threshold, and 4.5 periods per window. Dynamic spectral change was quantified using ΔF0, ΔF1, ΔF2, and ΔF3, computed as the difference between acoustic values at 75 and 25% of the vowel interval. Polygonal vowel space area (VSA) was computed from the F1–F2 coordinates of the five vowels, whereas vowel space dispersion (VSD) was calculated as the mean distance of individual vowel tokens from the group vowel-space centroid. No systematic measure-tracking errors requiring manual correction were identified. Where Praat failed to return a reliable acoustic value, the corresponding cell was left blank and treated as missing. No additional outlier-removal procedure was applied beyond the initial auditory/visual quality control and exclusion of problematic tokens.

**Figure 1:**
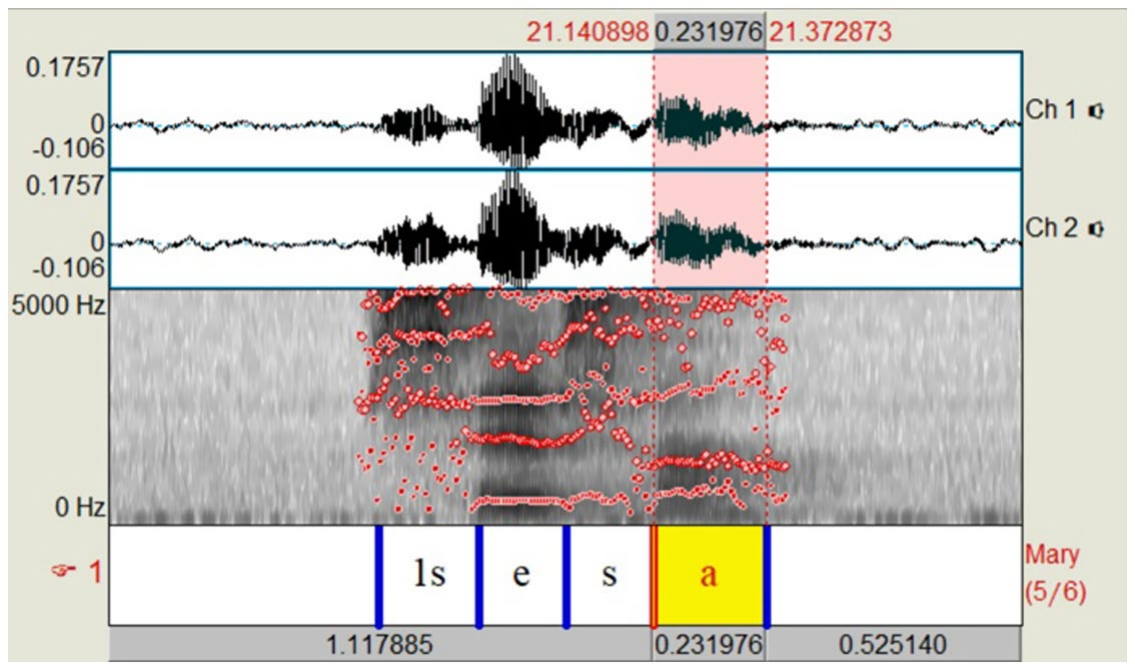
Example from the segmentation process.

To reduce between-speaker anatomical variation, F1, F2, and F3 were normalized using the Lobanov z-score method (Lobanov, 1971), and F0 was normalized using a semitone transformation relative to each speaker’s median F0. The remaining acoustic measures were standardized using *z*-scores to enhance cross-speaker comparability (Figure 1).

### 2.4 Statistical analyses

Group differences in VSA and VSD were analyzed using Bayesian regression models implemented in the brms package in R (R Core Team, 2026). The selected vowel-space and acoustic measures are widely used in phonetic and autism speech research, allowing direct comparison with previous studies examining ASD-related differences in vowel production and voice acoustics. Bayesian models were used because they are well-suited to modest sample sizes and allow effects to be estimated probabilistically rather than only through binary significance testing (Georgiou, 2024). They provide full posterior distributions, credible intervals, and estimates of uncertainty for each parameter, which is particularly useful in clinical and acoustic research where participant recruitment is often constrained. Bayesian modelling also allows prior information and hierarchical structure to be incorporated when appropriate, making it possible to obtain more stable and interpretable estimates of group differences in ASD-related speech measures.

Two separate models were estimated, one with VSA as the outcome and one with VSD as the outcome, with group entered as the fixed-effect predictor. For both models, weakly informative priors were specified for all regression parameters, including the intercept and residual variance, using a Student-t distribution with 3 degrees of freedom, mean 0, and scale 1 [student_t(3, 0, 1)]. These priors were chosen to provide regularization without imposing overly restrictive assumptions on the parameter estimates (see Georgiou, 2024). Inference regarding group differences in VSA and VSD was based on the posterior estimates and their 95% credible intervals (CrI).

To examine group differences in acoustic measures at the vowel level, we fitted a series of Bayesian mixed-effects regression models using the brms package in R. Separate models were estimated for each dependent variable: F0, F1, F2, F3, ΔF0, ΔF1, ΔF2, and ΔF3, duration, jitter, shimmer, HNR, and intensity. In each model, vowel, group, the vowel × group interaction, context, and stress were included as fixed effects, and participant was included as a random intercept to account for repeated observations within speakers. More complex random-effects structures, including more random slopes by participant, were considered because participants produced repeated tokens across these conditions. However, given the modest sample size and the large number of acoustic outcomes modeled separately, these models resulted in unstable estimation and/or convergence difficulties. Therefore, a more parsimonious random-intercept structure was retained to ensure model stability and interpretability.

Weakly informative Student-t priors, student_t(3, 0, 1), were specified for the regression coefficients, intercept, participant-level standard deviation, and residual standard deviation. Inference was based on posterior estimates and their 95% CrIs. To evaluate group differences within individual vowels, we conducted directed Bayesian hypothesis tests on linear combinations of model coefficients. These tests assessed whether the effect of group (ND relative to the reference group, namely ASD) was negative for each vowel category, based on the model parameterization and the relevant group and vowel × group terms. These tests were interpreted using the evidence ratio (ER) and posterior probability (PP), with ER ≥ 10 taken to indicate strong evidence for a group difference in the predicted direction and ER ≤ 0.1 taken to indicate strong evidence against the predicted direction. Posterior predictive checks were conducted for all models and indicated good model fit. Given the large number of acoustic outcomes and vowel-specific contrasts, these analyses were treated as exploratory and hypothesis-generating. ERs were interpreted as continuous measures of directional evidence and were considered together with the posterior estimates and 95% credible intervals reported in the tables, rather than as strict decision thresholds.

## 3 Results

### 3.1 Vowel space measures

**Figure 2:**
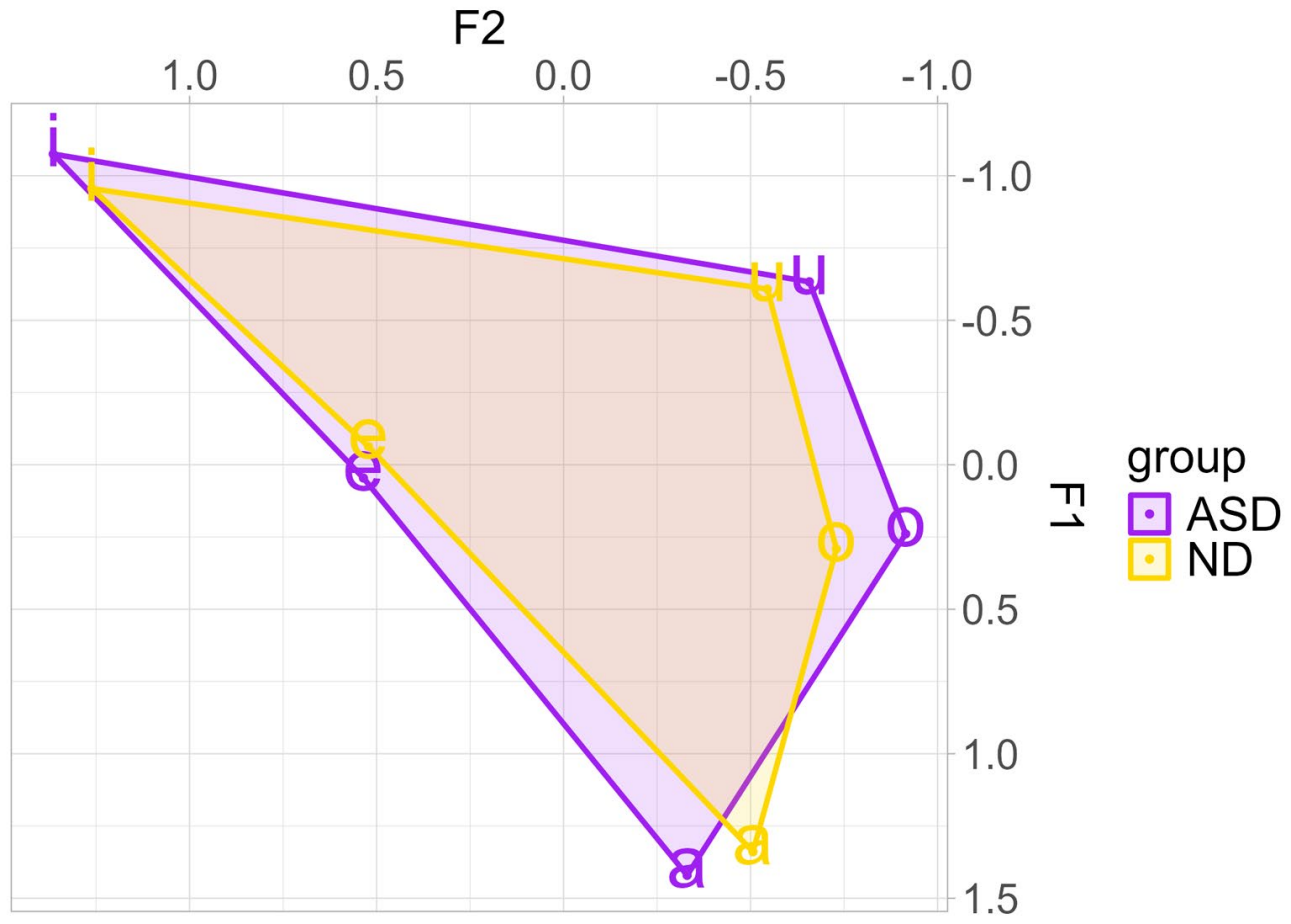
Two-dimensional vowel space (F1–F2) comparing productions of vowels for ASD (purple) and ND (yellow) groups. Vowel categories are connected to form polygonal spaces.

The two vowel space measures focused on the VSA and VSD. The Bayesian regression models indicated a credible effect of group on VSA. Compared to the ASD group (reference level), the ND group showed a lower VSA (*β* = −0.56, SE = 0.03, 95% CrI [−0.62, −0.50]). In addition, there was a credible effect of group on VSD, with the ND group showing lower VSD (*β* = −0.08, SE = 0.01, 95% CrI [−0.09, −0.07]) compared to the ASD group. Figure 2 shows the F1 × F2 vowel space of the ASD and ND groups.

### 3.2 Acoustic measures

**Figure 3:**
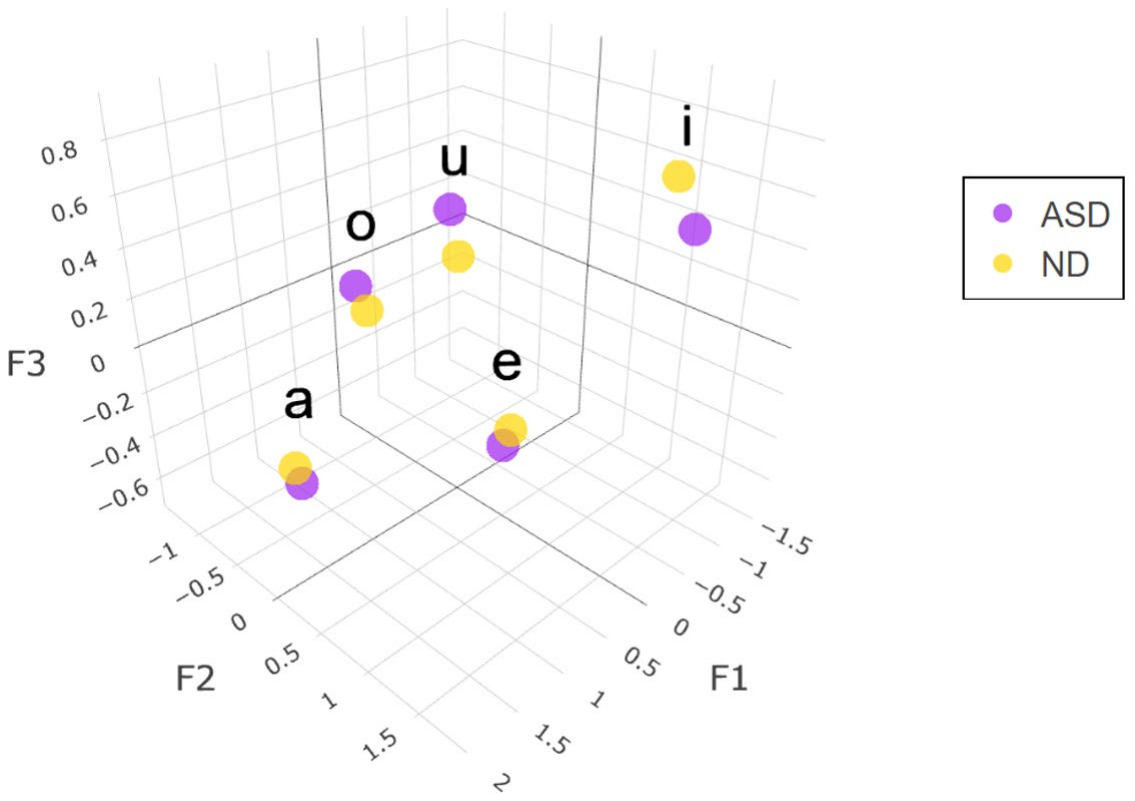
3D acoustic vowel space (F1–F2–F3) comparing productions of the five vowels between ASD (purple) and ND (yellow) groups.

The Bayesian models indicated that group effects were generally small and inconsistent across acoustic measures (see Table 3). For F0, there was no credible difference between groups. Similarly, no reliable group differences were observed for F1 or F3. In contrast, the ND group showed a small but credible decrease in F2 relative to the ASD group (*β* = −0.17, SE = 0.02, 95% CrI [−0.27, −0.08]). For dynamic measures, no credible group differences were observed for ΔF0, ΔF2, or ΔF3. However, there was weak evidence of a positive group effect for ΔF1 (*β* = 0.11, SE = 0.05, 95% CrI [0.00, 0.22]), suggesting slightly greater F1 movement in the ND group. Figure 3 illustrates the vowel acoustic space for F1, F2, and F3.

**Table 3:** The results of the Bayesian models regarding F0, formants, and dynamic trajectories.

Summary statistics							
Predictor	Estimate	Est. error	l − 95% CrI	u–95% CrI	Rhat bulk	ESS tail	ESS
F0							
Intercept	0.79	0.14	0.50	1.08	1.01	950	1,466
vowele	0.26	0.13	0.01	0.50	1	1,893	2,770
voweli	0.70	0.13	0.45	0.94	1	1,482	2,326
vowelo	0.35	0.13	0.10	0.59	1	2,047	2,548
vowelu	0.74	0.12	0.49	0.98	1	1,816	2,683
groupND	−0.32	0.20	−0.71	0.06	1.01	736	1,154
contextmedial	−0.01	0.06	−0.12	0.11	1	6,033	3,357
stressunstressed	−1.92	0.06	−2.03	−1.80	1	5,833	3,033
vowele:groupND	0.07	0.18	−0.28	0.43	1	1,802	2,511
voweli:groupND	0.04	0.18	−0.32	0.38	1	1,512	2,573
vowelo:groupND	−0.09	0.18	−0.45	0.26	1	1,985	2,636
vowelu:groupND	0.20	0.18	−0.14	0.55	1	1,802	2,664
F1							
Intercept	1.50	0.04	1.43	1.57	1	2,071	2,584
vowele	−1.37	0.05	−1.46	−1.28	1	2,273	2,507
voweli	−2.47	0.05	−2.57	−2.38	1	2,324	2,917
vowelo	−1.17	0.05	−1.26	−1.09	1	2,372	2,565
vowelu	−2.05	0.05	−2.14	−1.96	1	2,207	2,632
groupND	−0.07	0.05	−0.16	0.02	1	1,563	2,444
contextmedial	−0.04	0.02	−0.08	0.00	1	8,062	2,895
stressunstressed	−0.12	0.02	−0.16	−0.08	1	8,989	2,845
vowele:groupND	−0.03	0.06	−0.17	0.09	1	2,047	2,648
voweli:groupND	0.19	0.07	0.06	0.32	1	2,111	2,559
vowelo:groupND	0.13	0.07	0.00	0.25	1	2,180	2,592
vowelu:groupND	0.10	0.06	−0.03	0.23	1	1,906	2,467
F2							
Intercept	−0.41	0.04	−0.48	−0.34	1	2,729	3,144
vowele	0.87	0.05	0.77	0.96	1	2,819	3,497
voweli	1.68	0.05	1.59	1.78	1	2,918	3,075
vowelo	−0.58	0.05	−0.68	−0.49	1	3,054	3,104
vowelu	−0.32	0.05	−0.42	−0.23	1	2,749	3,198
groupND	−0.17	0.05	−0.27	−0.08	1	2,029	2,974
contextmedial	0.07	0.02	0.03	0.11	1	8,613	2,703
stressunstressed	0.09	0.02	0.05	0.13	1	10,696	2,737
vowele:groupND	0.16	0.07	0.03	0.29	1	2,726	3,079
voweli:groupND	0.07	0.07	−0.06	0.21	1	2,412	2,606
vowelo:groupND	0.36	0.07	0.23	0.49	1	2,698	2,984
vowelu:groupND	0.28	0.07	0.15	0.41	1	2,549	3,160
F3							
Intercept	−0.42	0.06	−0.53	−0.30	1	2,506	3,141
vowele	0.07	0.08	−0.08	0.21	1	2,594	3,140
voweli	0.93	0.08	0.78	1.08	1	3,059	3,369
vowelo	0.51	0.08	0.36	0.66	1	2,978	2,798
vowelu	0.75	0.08	0.60	0.90	1	2,739	2,994
groupND	0.00	0.07	−0.15	0.15	1	1,876	2,448
contextmedial	−0.04	0.03	−0.11	0.02	1	9,074	2,607
stressunstressed	−0.02	0.03	−0.09	0.05	1	8,375	2,703
vowele:groupND	0.05	0.10	−0.16	0.25	1	2,247	2,262
voweli:groupND	0.20	0.11	−0.01	0.41	1	2,751	3,116
vowelo:groupND	−0.06	0.10	−0.26	0.15	1	2,620	3,076
vowelu:groupND	−0.18	0.11	−0.39	0.04	1	2,494	3,023
ΔF0							
Intercept	0.16	0.15	−0.12	0.45	1	525	1,137
vowele	−0.11	0.11	−0.33	0.11	1	1,689	2,367
voweli	−0.20	0.11	−0.42	0.01	1	1,683	2,404
vowelo	−0.07	0.11	−0.28	0.14	1	1,603	1,504
vowelu	−0.20	0.11	−0.42	0.01	1	1,589	2,345
groupND	0.03	0.20	−0.37	0.41	1	570	1,059
contextmedial	−0.29	0.05	−0.39	−0.18	1	4,455	3,209
stressunstressed	−0.83	0.05	−0.93	−0.72	1	5,648	3,038
vowele:groupND	−0.04	0.16	−0.35	0.28	1	1,575	2,480
voweli:groupND	−0.10	0.16	−0.41	0.22	1	1,554	2,587
vowelo:groupND	0.17	0.15	−0.13	0.47	1	1,442	2,194
vowelu:groupND	0.05	0.16	−0.26	0.37	1	1,397	2,156
ΔF1							
Intercept	−0.22	0.04	−0.30	−0.13	1	2,794	3,178
vowele	0.11	0.05	0.00	0.22	1	2,825	3,325
voweli	0.28	0.05	0.17	0.38	1	2,893	3,263
vowelo	0.23	0.05	0.13	0.34	1	2,822	3,051
vowelu	0.38	0.06	0.27	0.49	1	2,998	3,320
groupND	0.11	0.05	0.00	0.22	1	2,049	2,743
contextmedial	0.08	0.03	0.04	0.13	1	9,814	2,762
stressunstressed	−0.05	0.03	−0.09	0.01	1	10,385	2,766
vowele:groupND	−0.02	0.08	−0.18	0.13	1	2,517	2,868
voweli:groupND	−0.12	0.08	−0.27	0.03	1	2,640	2,963
vowelo:groupND	−0.18	0.08	−0.32	−0.03	1	2,715	3,297
vowelu:groupND	−0.25	0.08	−0.40	−0.09	1	2,882	3,096
ΔF2							
Intercept	−0.02	0.04	−0.10	0.07	1	2,450	2,475
vowele	−0.01	0.05	−0.12	0.10	1	2,768	3,248
voweli	0.01	0.06	−0.10	0.12	1	2,565	2,571
vowelo	−0.02	0.06	−0.13	0.09	1	2,895	2,965
vowelu	0.09	0.06	−0.02	0.20	1	2,663	2,600
groupND	−0.01	0.05	−0.12	0.09	1	1,728	2,561
contextmedial	−0.08	0.02	−0.12	−0.03	1	10,696	2,668
stressunstressed	0.08	0.02	0.03	0.13	1	8,475	2,742
vowele:groupND	0.04	0.08	−0.11	0.19	1	2,602	3,014
voweli:groupND	0.15	0.08	0.00	0.30	1	2,393	3,005
vowelo:groupND	−0.01	0.08	−0.16	0.14	1	2,298	2,944
vowelu:groupND	−0.12	0.08	−0.27	0.03	1	2,496	2,774
ΔF3							
Intercept	−0.08	0.06	−0.20	0.04	1	2,587	2,635
vowele	0.01	0.08	−0.15	0.16	1	2,737	2,481
voweli	−0.14	0.08	−0.29	0.01	1	2,874	3,220
vowelo	0.02	0.08	−0.14	0.18	1	3,070	2,933
vowelu	0.09	0.08	−0.07	0.25	1	2,979	2,862
groupND	0.06	0.08	−0.10	0.22	1	2,021	2,742
contextmedial	0.05	0.04	−0.02	0.12	1	11,521	2,780
stressunstressed	0.12	0.04	0.05	0.20	1	9,274	2,019
vowele:groupND	−0.06	0.11	−0.28	0.16	1	2,638	3,229
voweli:groupND	−0.03	0.11	−0.25	0.20	1	2,510	3,016
vowelo:groupND	−0.10	0.11	−0.32	0.12	1	2,575	2,827
vowelu:groupND	−0.11	0.11	−0.33	0.12	1	2,801	2,854

The intercept terms are vowel = /a/ and group = ASD.

For the remaining acoustic measures, no credible group differences were observed. The ND group did not differ reliably from the ASD group in duration, jitter, shimmer, or intensity. For HNR, there was a negative trend for the ND group relative to the ASD group, but the credible interval included zero (see Table 4).

**Table 4:** Results of the Bayesian models regarding duration and voice quality measures.

Summary statistics							
	Estimate	Est. error	l − 95% CrI	u–95% CrI	Rhat bulk	ESS tail	ESS
Duration							
Intercept	0.40	0.10	0.20	0.60	1.01	672	1,303
vowele	−0.07	0.08	−0.22	0.07	1	1,593	2,476
voweli	−0.12	0.08	−0.27	0.04	1	1,683	2,695
vowelo	−0.11	0.08	−0.25	0.05	1	1,601	2,072
vowelu	−0.18	0.08	−0.33	−0.03	1	1,560	2,261
groupND	0.04	0.14	−0.22	0.31	1.01	633	1,440
contextmedial	−0.12	0.03	−0.19	−0.06	1	5,745	2,957
stressunstressed	−0.43	0.03	−0.49	−0.36	1	5,516	2,843
vowele:groupND	−0.09	0.11	−0.30	0.12	1	1,685	2,557
voweli:groupND	−0.16	0.11	−0.37	0.05	1	1,657	2,781
vowelo:groupND	−0.05	0.11	−0.27	0.16	1	1,566	2,546
vowelu:groupND	−0.05	0.11	−0.27	0.16	1	1,637	2,448
Jitter							
Intercept	−0.45	0.12	−0.69	−0.20	1.01	402	676
vowele	0.14	0.07	0.01	0.27	1	1,255	1,803
voweli	0.32	0.07	0.18	0.46	1.01	1,254	2,052
vowelo	0.03	0.07	−0.10	0.17	1	1,308	1,838
vowelu	0.12	0.07	−0.01	0.26	1	1,331	2,039
groupND	0.09	0.17	−0.25	0.43	1	453	862
contextmedial	−0.12	0.03	−0.18	−0.05	1	3,126	2,654
stressunstressed	0.68	0.03	0.62	0.74	1	3,424	2,873
vowele:groupND	−0.06	0.10	−0.25	0.13	1	1,177	2,169
voweli:groupND	−0.07	0.10	−0.27	0.12	1.01	1,189	1,955
vowelo:groupND	0.09	0.10	−0.10	0.28	1	1,318	2,092
vowelu:groupND	0.00	0.10	−0.18	0.19	1	1,359	2,092
Shimmer							
Intercept	−0.18	0.12	−0.42	0.05	1.01	429	758
vowele	0.05	0.07	−0.10	0.19	1	1,142	2,074
voweli	0.05	0.07	−0.09	0.19	1	1,149	1,778
vowelo	−0.04	0.07	−0.18	0.10	1.01	1,130	2,452
vowelu	0.01	0.07	−0.12	0.16	1.01	995	2,302
groupND	0.15	0.17	−0.17	0.48	1.01	478	890
contextmedial	−0.21	0.03	−0.28	−0.15	1	3,731	2,825
stressunstressed	0.45	0.03	0.39	0.52	1	3,571	2,919
vowele:groupND	−0.06	0.10	−0.26	0.14	1	1,162	2,071
voweli:groupND	0.11	0.10	−0.10	0.32	1	1,248	2,240
vowelo:groupND	−0.01	0.10	−0.20	0.19	1	1,149	2,259
vowelu:groupND	−0.10	0.10	−0.30	0.10	1.01	1,149	2,035
HNR							
Intercept	−0.02	0.15	−0.31	0.27	1.01	404	788
vowele	0.31	0.06	0.20	0.42	1	1,418	1,924
voweli	0.85	0.06	0.75	0.96	1	1,466	2,065
vowelo	0.40	0.05	0.30	0.51	1	1,463	2,388
vowelu	0.94	0.06	0.83	1.05	1	1,267	1,675
groupND	−0.36	0.20	−0.74	0.02	1.01	487	926
contextmedial	0.08	0.03	0.03	0.13	1	2,311	2,383
stressunstressed	−0.67	0.03	−0.72	−0.62	1	2,750	2,173
vowele:groupND	−0.01	0.08	−0.17	0.14	1	1,349	1,514
voweli:groupND	0.02	0.08	−0.14	0.18	1	1,443	1,417
vowelo:groupND	−0.03	0.08	−0.18	0.13	1	1,538	1,863
vowelu:groupND	−0.05	0.08	−0.20	0.11	1	1,334	1,562
Intensity							
Intercept	0.48	0.21	0.07	0.89	1.01	936	1,383
vowele	−0.15	0.03	−0.22	−0.09	1	2,566	2,758
voweli	−0.28	0.03	−0.35	−0.21	1	2,548	2,679
vowelo	−0.03	0.03	−0.10	0.03	1	2,658	2,585
vowelu	−0.21	0.03	−0.27	−0.14	1	2,672	2,913
groupND	−0.26	0.29	−0.82	0.31	1.01	725	1,263
contextmedial	0.17	0.02	0.15	0.20	1	4,262	2,603
stressunstressed	−0.59	0.01	−0.62	−0.56	1	4,340	2,462
vowele:groupND	0.07	0.05	−0.02	0.16	1	2,560	2,746
voweli:groupND	0.02	0.05	−0.07	0.11	1	2,389	2,862
vowelo:groupND	−0.02	0.05	−0.11	0.07	1	2,697	2,752
vowelu:groupND	0.03	0.05	−0.07	0.12	1	2,641	2,641

The intercept terms are vowel = /a/ and group = ASD.

**Figure 4:**
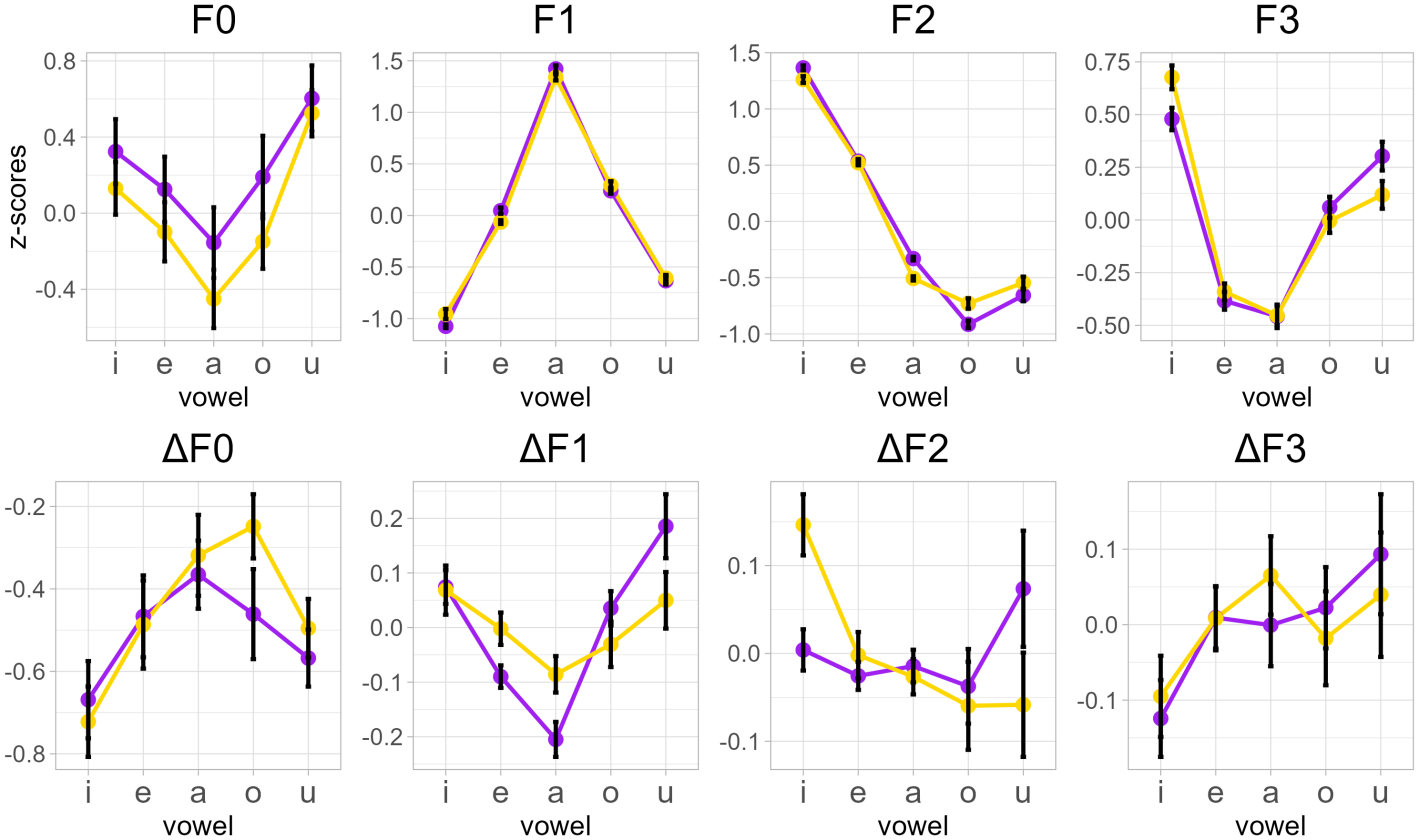
Vowel-specific acoustic measures (z-scores) for ASD (purple) and ND (yellow) groups across the following parameters: F0, F1, F2, F3, ΔF0, ΔF1, ΔF2, and ΔF3. Points represent mean values for each vowel, with error bars indicating variability (±SE).

Evidence ratios indicated strong support for several vowel-specific group differences. For F0, there was strong evidence that the ND group had lower values than the ASD group for /i/ (ER = 11.62, PP = 0.92), /a/ (ER = 19.30, PP = 0.95), and /o/ (ER = 55.34, PP = 0.98). For F1, strong evidence indicated lower values in the ND group for /e/ (ER = 87.89, PP = 0.99) and /a/ (ER = 17.26, PP = 0.95), while there was strong evidence in the opposite direction for /i/ (ER = 0.01, PP = 0.01). For F2, strong evidence showed lower values for the ND group in /i/ (ER = 56.14, PP = 0.98) and /a/ (ER = ∞, PP = 1.00), and higher values in the ND group for /o/ (ER = 0.00, PP = 0.00) and /u/ (ER = 0.01, PP = 0.01). For F3, strong evidence indicated lower values in the ND group for /u/ (ER = 77.43, PP = 0.99) and higher values for /i/ (ER = 0.00, PP = 0.00). No strong evidence was observed for ΔF0. For ΔF1, strong evidence indicated lower values in the ND group for /u/ (ER = 110.11, PP = 0.99) and higher values for /a/ (ER = 0.02, PP = 0.02) and /e/ (ER = 0.06, PP = 0.02). For ΔF2, strong evidence showed higher values in the ND group for /i/ (ER = 0.01, PP = 0.01) and lower values for /u/ (ER = 136.93, PP = 0.99). No strong evidence was observed for ΔF3. These results are presented in Table 5. Figure 4 illustrates the differences between the ASD and ND groups in terms of F0, formants, and dynamic trajectories.

**Table 5:** Results of hypothesis testing for F0, formants, and dynamic trajectories.

ND < ASD	Estimate	Est. error	CrI. lower	CrI. upper	Evid. ratio	Post prob.
F0						
i	−0.28	0.20	−0.62	0.05	11.62	0.92
e	−0.24	0.20	−0.56	0.07	8.30	0.89
a	−0.32	0.20	−0.64	0.00	19.30	0.95
o	−0.41	0.20	−0.75	−0.08	55.34	0.98
u	−0.11	0.20	−0.44	0.21	2.46	0.71
F1						
i	0.12	0.05	0.04	0.20	0.01	0.01
e	−0.11	0.05	−0.19	−0.03	87.89	0.99
a	−0.07	0.05	−0.15	0.00	17.26	0.95
o	0.05	0.05	−0.03	0.13	0.17	0.14
u	0.03	0.05	−0.05	0.10	0.38	0.28
F2						
i	−0.10	0.05	−0.18	−0.02	56.14	0.98
e	−0.01	0.05	−0.09	0.07	1.60	0.62
a	−0.17	0.05	−0.25	−0.09	Inf	1.00
o	0.18	0.05	0.10	0.26	0.00	0.00
u	0.11	0.05	0.03	0.18	0.01	0.01
F3						
i	0.20	0.08	0.07	0.33	0.00	0.00
e	0.04	0.07	−0.08	0.16	0.38	0.27
a	0.00	0.07	−0.13	0.12	1.05	0.51
o	−0.06	0.07	−0.18	0.06	4.01	0.80
u	−0.18	0.08	−0.31	−0.05	77.43	0.99
ΔF0						
i	−0.07	0.20	−0.39	0.26	1.79	0.64
e	−0.02	0.20	−0.35	0.32	1.15	0.54
a	0.03	0.20	−0.30	0.35	0.79	0.44
o	0.20	0.20	−0.14	0.52	0.19	0.16
u	0.07	0.20	−0.26	0.40	0.54	0.35
ΔF1						
i	−0.01	0.05	−0.10	0.08	1.17	0.54
e	0.09	0.06	0.00	0.18	0.06	0.06
a	0.11	0.05	0.02	0.20	0.02	0.02
o	−0.07	0.05	−0.15	0.03	7.85	0.89
u	−0.13	0.06	−0.23	−0.04	110.11	0.99
ΔF2						
i	0.14	0.06	0.05	0.23	0.01	0.01
e	0.02	0.06	−0.07	0.12	0.48	0.32
a	−0.01	0.05	−0.10	0.08	1.36	0.58
o	−0.02	0.06	−0.11	0.07	1.80	0.64
u	−0.13	0.06	−0.22	−0.04	136.93	0.99
ΔF3						
i	0.03	0.08	−0.10	0.16	0.57	0.36
e	0.00	0.08	−0.14	0.13	1.08	0.52
a	0.06	0.08	−0.07	0.19	0.29	0.22
o	−0.04	0.08	−0.17	0.09	2.33	0.70
u	−0.05	0.08	−0.19	0.09	2.90	0.74

**Figure 5:**
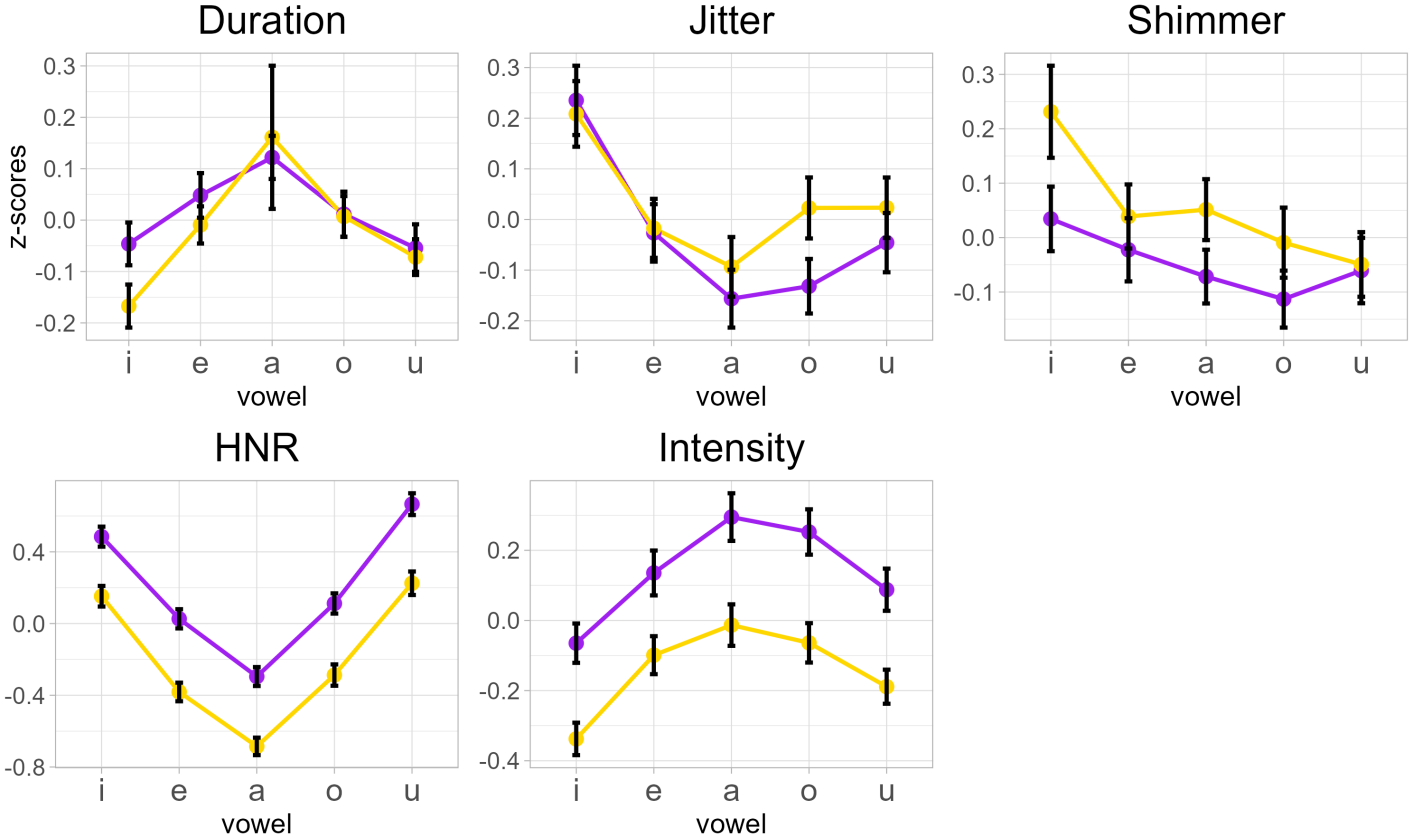
Vowel-specific acoustic measures (z-scores) for ASD (purple) and ND (yellow) groups across five parameters: duration, jitter, shimmer, HNR, and intensity. Points represent mean values for each vowel, with error bars indicating variability (±SE).

Although the global HNR group effect did not exclude zero, the exploratory vowel-specific contrasts showed a consistent directional pattern, with lower estimated HNR in the ND group than in the ASD group across all vowels: /i/ (ER = 18.61, PP = 0.95), /e/ (ER = 27.17, PP = 0.96), /a/ (ER = 28.85, PP = 0.97), /o/ (ER = 36.04, PP = 0.97), and /u/ (ER = 46.62, PP = 0.98). This difference reflects the model parameterization: the global group coefficient represents the group contrast at the reference vowel, whereas the vowel-specific contrasts combine the group coefficient with the relevant vowel × group interaction terms. No strong evidence of group differences was found for duration, jitter, shimmer (except /i/), or intensity. The results are shown in Table 6. Figure 5 illustrates the differences between the ASD and ND groups in terms of duration, voice quality measures, and intensity.

**Table 6:** Results of hypothesis testing for duration and voice quality measures.

ND < ASD	Estimate	Est. error	CrI. lower	CrI. upper	Evid. ratio	Post prob.
Duration						
i	−0.12	0.14	−0.34	0.10	4.35	0.81
e	−0.05	0.13	−0.28	0.16	1.81	0.64
a	0.04	0.14	−0.19	0.26	0.63	0.39
o	−0.01	0.13	−0.23	0.20	1.06	0.52
u	−0.01	0.13	−0.23	0.20	1.09	0.52
Jitter						
i	0.03	0.17	−0.26	0.30	0.75	0.43
e	0.04	0.17	−0.26	0.32	0.68	0.40
a	0.09	0.17	−0.19	0.37	0.40	0.28
o	0.19	0.17	−0.09	0.46	0.16	0.14
u	0.10	0.17	−0.19	0.38	0.39	0.28
Shimmer						
i	0.26	0.17	−0.01	0.54	0.06	0.06
e	0.09	0.17	−0.18	0.37	0.38	0.28
a	0.15	0.17	−0.11	0.43	0.21	0.18
o	0.14	0.17	−0.13	0.42	0.25	0.20
u	0.05	0.17	−0.22	0.33	0.58	0.37
HNR						
i	−0.33	0.20	−0.67	0.00	18.61	0.95
e	−0.37	0.20	−0.70	−0.04	27.17	0.96
a	−0.36	0.20	−0.69	−0.03	28.85	0.97
o	−0.39	0.20	−0.72	−0.05	36.04	0.97
u	−0.41	0.20	−0.73	−0.08	46.62	0.98
Intensity						
i	−0.24	0.29	−0.71	0.23	4.12	0.80
e	−0.19	0.29	−0.65	0.27	2.96	0.75
a	−0.26	0.29	−0.72	0.20	4.57	0.82
o	−0.28	0.29	−0.75	0.18	5.36	0.84
u	−0.23	0.29	−0.70	0.23	3.83	0.79

## 4 Discussion

The present study examined vowel production in Cypriot Greek speakers with ASD and ND peers using a controlled pseudoword-reading paradigm and a broad acoustic battery spanning static spectral measures, dynamic spectral change, temporal structure, vowel-space organization, and voice-quality indices. Three main findings emerged, which broadly support the hypotheses formulated earlier. First, the ASD group showed a larger vowel-space area and greater vowel-space dispersion than the ND group. Second, group differences at the level of individual acoustic parameters were mostly selective rather than global, with the clearest effects concentrated primarily in F0 and formant frequencies, and secondarily in the dynamic formant measures ΔF1 and ΔF2, and with many effects localized to particular vowels rather than extending uniformly across the system. Third, among the voice-quality measures, the most consistent vowel-specific pattern was that the ND group tended to show lower HNR than the ASD group across vowels, although the global HNR group effect was uncertain, whereas duration, jitter, shimmer, and intensity did not yield robust overall group differences. Collectively, these findings argue against a unitary notion of an “autistic voice” and instead support a more differentiated account in which autism-related differences in speech are distributed unevenly across acoustic domains, vowels, and task conditions (Asghari et al., 2021; Fusaroli et al., 2017, 2022; Ma et al., 2024; Van Santen et al., 2010). This study advances autism speech research by showing, in a tightly controlled framework and an underrepresented language variety, that autism-related speech differences are concentrated in vowel-space organization, selected spectral properties, and HNR rather than uniformly distributed across all acoustic domains.

A central contribution of the present study is the finding that the ASD group occupied a larger and more dispersed acoustic vowel space than the ND group, being consistent with our initial hypothesis for vowel-space differences between the two groups. This pattern is noteworthy because previous research has identified ASD–ND differences in vowel-space organization, but has not converged on a single direction of effect. Lyakso et al. (2016), for example, reported larger vowel formant triangles in children with ASD, a pattern broadly compatible with the present findings. By contrast, Kissine et al. (2021) found the opposite tendency in autistic adults, who showed reduced dispersion and greater phonetic stability, interpreted as stronger attraction toward native vowel targets. The present results, therefore, align more closely with Lyakso et al. (2016) and suggest that, at least in Cypriot Greek and under the elicitation conditions used here, ASD speakers may realize vowel categories with greater acoustic spread and less centralized clustering than ND speakers. One possible interpretation is not simply that the task reduced lexical and pragmatic demands, but that it allowed segmental implementation itself to become more visible. Under these conditions, the larger vowel space may reflect a tendency toward more extreme realization of some vowel targets, greater trial-to-trial variability in articulatory positioning, or weaker compression of productions around category prototypes. On this view, the key difference may lie less in whether ASD speakers can produce the relevant vowel categories and more in how consistently and tightly those categories are organized in acoustic space.

The absence of a robust overall group effect for mean F0 also deserves comment. At first glance, this may seem surprising given the long-standing association between autism and atypical prosody (e.g., Asghari et al., 2021), but it is, in fact, compatible with current syntheses of the field. More specifically, the present data align with the results of Arciuli and Bailey (2019), who indicated no differences in F0 between autistic and neurotypical children in vowel acoustics. Yet, vowel-specific tests still showed evidence of F0 differences for three vowels (/i a o/), with the clearest effect in /o/. This suggests that autistic speech in the present data was not simply globally higher-pitched or lower-pitched. Rather, pitch differences, when present, appear to have been localized. Furthermore, the overall ASD–ND difference in F2 is in line with prior findings suggesting that formant structure may help distinguish autistic from neurotypical speech (Mohanta and Mittal, 2022; Sadiq et al., 2025). The particularly broad involvement of F2 suggests that group differences may be related in part to front–back acoustic differentiation. However, the statistical models did not indicate broad, uniform group effects across all formants, as vowel-specific hypothesis tests revealed a selective pattern of differences across the vowel system. This result corroborates our initial hypothesis for vowel-specific effects and is partly consistent with Mohanta and Mittal (2022), who also reported differences in some vowels between ASD and ND children, although these were observed in F4. In this study, evidence for group differences emerged in F1 for three vowels (/i e a/) and in F2 for four vowels (/i a o u/), with more limited effects in F3 (/i u/). This pattern is theoretically meaningful because several of the affected vowels are corner or peripheral vowels, which contribute disproportionately to vowel-space geometry and to the acoustic distinctiveness of the system as a whole. The dynamic results extend this picture, since although there was little evidence for systematic differences in ΔF0 or ΔF3, clearer effects emerged in ΔF1 for /e a u/ and in ΔF2 for /i u/; this indicates that within-vowel spectral movement also contributed to some extent to group differentiation. In sum, these findings suggest that the ASD group differed from the ND group not only in static vowel targets but also in aspects of within-vowel acoustic change over time. This interpretation is consistent with emerging work showing that autism-related speech differences are not well captured by a single dimension, but may instead be distributed across articulation, spectral structure, and dynamic acoustic patterning (Briend et al., 2023; Patel et al., 2020).

A further notable outcome was the lack of robust overall group differences in duration, jitter, shimmer, and intensity, as projected in our hypotheses. In light of previous work, this pattern is not entirely surprising. For duration, the vowel-level literature remains limited and tends to favor null or near-null findings under structured elicitation conditions (Arciuli and Bailey, 2019). The present findings, therefore, add to a growing indication that vowel timing, at least in controlled tasks, may often be broadly comparable across ASD and ND speakers. The null findings for jitter and shimmer can also be interpreted cautiously in relation to the existing literature. Although Kissine et al. (2021) reported lower jitter and shimmer in autistic adults, broader evidence suggests that these measures are not consistently replicated and may be highly sensitive to methodological factors (MacCallum et al., 2011; Yousef and Hunter, 2024). The absence of such differences in the present study – both global and vowel-specific – therefore supports the view that such measures may not generalize reliably across samples and paradigms, even when some individual studies report significant effects. A similar interpretation applies to intensity, for which no within-vowel differentiations between the two groups emerged (see also Arciuli and Bailey, 2019; Sadiq et al., 2025). The present null result is therefore consistent with the possibility that intensity is not a robust differentiating feature of vowel production in ASD under the current elicitation conditions.

Perhaps the most intriguing result concerns HNR, for which the ND group showed lower values than the ASD group across all vowels. In many clinical voice contexts, higher HNR is interpreted as reflecting greater acoustic periodicity and lower noise in the signal. On the surface, this may seem counterintuitive relative to work linking autism with hoarser or creakier voice qualities, or with reduced harmonicity in spontaneous speech. For example, Bone et al. (2014) reported that in spontaneous prosody, children’s speech associated with ASD tended to show decreased HNR, while Fusaroli et al. (2022) identified increased hoarseness and creakiness as part of a minimal cross-linguistic acoustic profile. Our result is consistent with Briend et al. (2023), who also found higher HNR in children with ASD during a nonword repetition task. One possible explanation for our finding is that the higher HNR observed in the ASD group reflects a more controlled or constrained production style during the elicited word task. Because predictable task structure can facilitate performance consistency in autistic adults, it is plausible that the repetition-based format supported more stable and less noisy phonatory output, resulting in higher HNR (Lacroix et al., 2025). Taking into account that HNR is highly sensitive to recording quality, segmentation, and phonatory context, the present finding should be interpreted as a task-specific acoustic pattern rather than a direct clinical index of “better” or “worse” voice quality. Also, since HNR and intensity are not entirely independent acoustically, some vowel-specific differences in intensity were also expected. Although the descriptive statistics visually suggested some vowel-level intensity variation between groups, the statistical models did not indicate robust or consistent intensity differences comparable to those observed for HNR. This suggests that the HNR pattern is unlikely to be explained solely by greater vocal intensity or loudness in the ASD group. However, some degree of interaction between phonatory stability and vocal intensity cannot be excluded and warrants more targeted investigation in future work.

Taken together, these patterns highlight why developmental stage, elicitation method, and language must be considered when comparing ASD speech findings across studies. Because the present study examined adults, the expanded vowel space observed here should not be interpreted simply as the adult continuation of child patterns, since children’s vowel spaces are still shaped by vocal-tract growth and developing speech-motor control. In terms of methodology, the findings align most closely with controlled production studies: the expanded vowel space is consistent with Lyakso et al. (2016), and the selective formant effects partly align with Mohanta and Mittal (2022), while the higher HNR pattern is consistent with Briend et al. (2023). However, the present findings differ from Kissine et al. (2021), who found reduced vowel dispersion in French-speaking autistic adults, and from Bone et al. (2014), who reported reduced HNR in spontaneous speech. In linguistic terms, the Cypriot Greek findings resemble Russian and English evidence in showing expanded or formant-differentiated vowel production, but differ from French evidence showing reduced dispersion. This suggests that ASD-related acoustic differences are not expressed identically across languages. Rather, the acoustic manifestation of ASD may depend partly on the structure of the ambient vowel system, including vowel inventory size, vowel-space density, and the degree of peripheral vowel contrast. Thus, in Cypriot Greek, a five-vowel system with relatively clear peripheral vowel categories, ASD-related differences appear to be expressed primarily through expanded and vowel-specific acoustic implementation rather than through a uniform shift across all speech measures.

The findings do not support the idea of a single acoustic phenotype of autism. Instead, they suggest that autistic speech may involve differences in how speech-motor, auditory, and phonatory systems organize fine-grained phonetic output. The expanded vowel space in the ASD group may reflect more extreme vowel target realization, greater articulatory dispersion, or reduced compression around category prototypes. Such an interpretation is compatible with broader evidence that autism is often associated with differences in motor coordination and motor planning (Fournier et al., 2010). It is also compatible with predictive-processing accounts of autism, according to which differences in prediction, sensory precision, or feedback-based updating may affect perception–action coordination (Sinha et al., 2014). In this sense, vowel-production differences may represent one behavioral manifestation of altered coordination among perception, motor planning, and speech execution. In addition, at the mechanistic level, several pathways could plausibly contribute to these acoustic patterns. Speech production depends on interactions among auditory feedback, somatosensory feedback, motor planning, and feedforward control (Guenther, 2016). Differences in these systems could affect articulatory target selection, vowel-space dispersion, or the stability of formant trajectories. In autism, broader motor and sensorimotor differences have been repeatedly documented, including differences in coordination, timing, and adaptive motor control (Gowen and Hamilton, 2013). HNR differences may additionally reflect task-specific phonatory control, including the regularity of vocal-fold vibration during structured reading. However, the present acoustic data cannot identify neural or laryngeal mechanisms directly. Therefore, these findings should be interpreted as behavioral acoustic evidence consistent with altered speech-motor and phonatory organization, not as direct evidence for a specific neurobiological cause of ASD.

The social relevance of these findings is also important. Speech and voice characteristics can influence first impressions and social judgments, including perceptions of naturalness, affect, engagement, or communicative intent (Sasson et al., 2017). Even subtle differences in vowel-space structure, pitch, or voice quality may therefore contribute to how autistic speech is perceived by listeners, especially when combined with prosodic, pragmatic, or interactional cues. However, these differences should not automatically be treated as deficits requiring remediation. Contemporary neurodiversity-informed approaches emphasize that intervention should support communication, autonomy, comfort, and participation rather than aiming to normalize autistic behavior or make autistic individuals appear neurotypical (Bottema-Beutel et al., 2021). The present findings may therefore be most useful for characterization, awareness, and individualized support, not for pathologizing autistic voice quality.

## 5 Conclusion

### 5.1 Implications

The present study provides new evidence that vowel production in Cypriot Greek differs between ASD and ND speakers in specific, non-uniform ways. The most robust differences were not global shifts in mean pitch, duration, or perturbation, but rather expanded vowel-space structure, selective pitch and formant contrasts, dynamic spectral differences, and a pattern of higher HNR across vowels in the ASD group. These findings strengthen the case for moving beyond broad labels and toward a more fine-grained account of autistic speech, one that recognizes the joint roles of articulation, phonation, vowel-specific properties, task structure, and language-specific phonetic organization. In this way, the study contributes both to the cross-linguistic literature on autism acoustics and to the broader effort to identify speech features that are sufficiently robust, interpretable, and context-sensitive to support future descriptive research and provide a foundation for future translational studies examining potential screening-related applications. It also broadens the empirical basis for theories of autism-related speech by suggesting that acoustic distinctions may be shaped partly by the structure of the ambient vowel system.

### 5.2 Limitations and future work

The findings should be interpreted in light of several limitations. The sample was necessarily modest due to practical constraints (e.g., small population of Cyprus, restricted timeframes for data collection), and although the Bayesian framework is well-suited to smaller datasets, the present results still require replication in larger cohorts. In addition, the task was intentionally constrained; accordingly, the findings cannot be assumed to generalize directly to spontaneous speech, affective prosody, or conversation. This point is especially relevant given evidence that interactional synchrony and discourse context can reveal differences not visible in decontextualized speech (Ochi et al., 2019), and that listener impressions of autistic speech may depend on combinations of cues not well captured by single acoustic measures (Patel et al., 2020). Due to the methodological limitations, the results also cannot be generalized to children, minimally verbal autistic individuals, autistic speakers with intellectual disability, or speakers of other languages/dialects. The male-skewed sample also limits conclusions about sex/gender differences. A further limitation is that the study centered on vowel acoustics and did not model consonantal timing, speech rhythm, cepstral measures, or articulatory variability across repetitions. These domains are promising targets for future work, especially in light of recent research suggesting that speech consistency, coordination, and repetition-based measures may uncover additional dimensions of motor-acoustic organization in autism (Maffei et al., 2025). Finally, the analyses relied mainly on group-level averages and static or two-time-point acoustic measures. Because speech production is dynamic, this approach may have missed informative within-token trajectories and speaker-specific variability.

Correspondingly, future research should address these limitations in five ways. First, the findings should be replicated in larger cohorts to assess the stability and generalizability of the observed acoustic patterns. Second, future studies should test whether the vowel-space and HNR effects persist across less constrained contexts, including spontaneous speech, affective prosody, and conversation. Third, research should include more diverse participant groups, including children, minimally verbal autistic individuals, autistic speakers with intellectual disability, speakers of other languages or dialects, and more balanced sex/gender samples. Fourth, future work should extend beyond vowel acoustics by examining consonantal timing, speech rhythm, cepstral measures, and articulatory variability across repetitions. Fifth, because speech production is dynamic, future analyses should move beyond group-level averages and static or two-time-point measures by modelling within-token trajectories, trial-to-trial variability, and speaker-specific acoustic profiles.

## Data Availability

The raw data supporting the conclusions of this article will be made available by the authors, without undue reservation.
